# Breast Tumor Microcalcification Induced by Bone Morphogenetic Protein-2: A New Murine Model for Human Breast Tumor Diagnosis

**DOI:** 10.1155/2018/2082154

**Published:** 2018-11-11

**Authors:** Asghar Hajibeigi, Khaled Nasr, Durga Udayakumar, Kien Nham, Robert E. Lenkinski

**Affiliations:** ^1^Department of Radiology, UT Southwestern Medical Center, Dallas, TX 75390, USA; ^2^Advanced Imaging Research Center, UT Southwestern Medical Center, Dallas, TX 75390, USA

## Abstract

Widespread use of screening mammography has recently increased the detection of breast microcalcifications. These nonpalpable microcalcifications with specific features in breast tissues are clinically considered an early indicator of breast carcinoma. Our goal in this study was to develop a murine breast microcalcification model for optimizing *in vivo* imaging. Recombinant human BMP-2 was expressed in *E. coli*, and the purified bioactive protein was used as inducing factor for the production of breast microcalcifications in a murine animal model. Syngeneic breast tumors were obtained by injection of MDA-MB-231 human breast cancer cells with Matrigel into the mammary fat pad of female nude mice. Different doses of bioactive rhBMP-2 were administered either as single or multiple intraperitoneal injections or directly into tumor on a weekly basis. Three weeks after the first injection of rhBMP-2, the microcalcification of breast tumor was detected by microcomputed tomography followed by intravenous injection of radiotracer [^18^F] Sodium fluoride for positron emission tomography imaging. Our findings indicate that rhBMP-2 induced microcalcifications of breast tumor by both systemic and direct injection of rhBMP-2 into tumors in a dose-dependent manner. Although little is known about the molecular mechanism of microcalcification, here we report a new murine model of human breast tumor induced microcalcification by rhBMP-2 to optimize in vivo imaging methods and to study the role of BMP-2 as a mediator of pathological mineralization and bone-like microcalcification formation in breast tumor. This BMP-2-induced microcalcification model may allow us to discriminate the type of microcalcification in tumors and to perform quantitative analysis on the calcification as a new detection strategy for early identification of pathological mineralization of breast tissues in women.

## 1. Introduction

Breast cancer is a disease of high prevalence among women in the western and industrialized countries. It represents 15% of all new cancer cases in the US [1]. Mammography is currently the gold standard for the detection of early, clinically occult breast cancer [2, 3]. One feature of particular diagnostic significance is the presence of microcalcifications on the mammogram [2], and frequently these calcifications are the only mammographic feature that indicates the presence of a malignant lesion. Up to 50% of all nonpalpable breast cancers are detected solely through microcalcifications observed on mammography, and up to 93% of cases of ductal carcinoma in situ (DCIS) present with microcalcifications [4]. Haka et al. [5] applied Raman spectroscopy to analyze the chemical composition of microcalcifications occurring in benign and malignant lesions in the human breast and identified two major types of microcalcifications. Type I, calcium oxalate dihydrate crystals are seen most frequently in benign ductal cysts and are rarely found in foci of carcinoma, whereas the type II calcium phosphate deposits (in the form of hydroxyapatite (HA)) are most often seen in proliferative lesions, including carcinomas [6]. Type I microcalcifications were diagnosed as benign, whereas type II were subdivided into benign and malignant ducts with a sensitivity of 88% and a specificity of 93% [4]. The malignant HA microcalcifications can present in a variety of different shapes (e.g., needle like or pleomorphic) and sizes (ranging from 40 to several hundred microns). There is increasing evidence that the presence of HA microcalcifications correlates with more aggressive disease, correlating with lymph invasion and Her-2 expression [7, 8]. Castronovo and Bellahcene [9] have suggested that the deposition of HA in breast cancer is an active process rather than mineralization of cellular debris or necrotic material.

Our group has been involved in developing a variety of imaging agents that are specifically targeted to selectively visualizing HA-based microcalcifications in breast cancer [10–13]. At the time we started this effort, we found that there were no rodent models that had predicable, radiographically, detectable microcalcifications to optimize our imaging efforts. We established two models in rats that fulfilled these criteria [10, 14] based on the osteogenic effects of bone morphogenetic protein-2 (BMP-2). We chose BMP-2 because it is a member of the transforming growth factor-*β* superfamily in osteogenesis and vascular calcification has been well characterized. It has been shown that the expression of BMP-2 both *in vitro* and *in vivo* can enhance the osteoblast and osteoblast-like cells differentiation and stimulate matrix mineralization [15, 16]. Furthermore, studies have shown that the extent of microcalcifications in breast correlates with an increase of BMP-2 levels in serum [14, 15]. There have been two recent reports assessing noninvasive imaging of the presence of microcalcifications in a flank murine model using implanted human derived MDA-MB-231 breast cancer cells [17, 18]. However, these microcalcifications were microscopic and difficult to detect using conventional noninvasive *in vivo* multimodality imaging methods. The aim of the present study was to extend our approach and develop orthotropic microcalcified breast tumors using human derived cell lines in murine models using rhBMP-2 as an inducer. This microcalcification model will play an important role in testing and optimization of targeted contrast agents for the early detection of breast lesions using *in vivo* multimodality imaging.

## 2. Materials and Methods

### 2.1. Bacterial Expression of Recombinant BMP-2 and Its Purification

**Figure 1:**
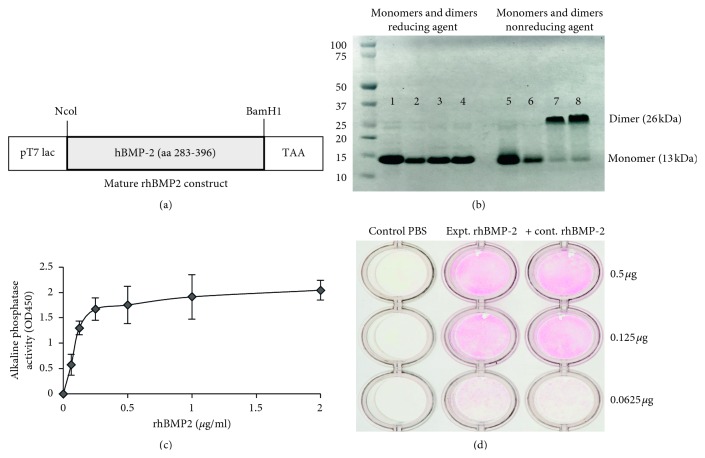
(a) Expression, purification, and bioactivity of rhBMP-2: the bacterial expression construct encoding amino acids 288–396 was used for expression of rhBMP-2 (modified from [14]). Following the renaturation and purification using heparin column, 0.5 *μ*g of eluted protein samples (monomer and dimer) was analyzed by NuPAGE gel electrophoresis as shown (b). Lanes 1–4 with reducing agent and lanes 5–8 without reducing agent. C2C12 cells were treated with various doses of purified rhBMP-2. After 72 h in culture, the alkaline phosphatase activity was measured from cell lysates shown in (c) and directly by colorimetric assay (d).

The *E. coli* strain Bl21 (DE3) harboring the mature human BMP-2 construct (Figure 1) kindly provided by Liu et al. [14] was cultivated in Luria Bertani (LB) broth medium supplemented with ampicillin (100 *μ*g/ml) at 37°C overnight. When the OD_600_ of the culture reached about 0.6–0.8, the expression of rhBMP-2 was induced by the addition of 1 mM isopropyl-b-*D*-thiogalactopyranoside (IPTG) for 4 h. For the extraction of inclusion bodies, the cells were harvested by centrifugation at 5000 rpm, cell pellets washed twice with 50 mM sodium phosphate buffer (pH 7.0) and then resuspended in 20 mM Tris-HCl (pH 8.5), 0.5 mM EDTA, and 2% Triton X100. Solubilized inclusion bodies were sonicated twice for 2 min in ice followed by 30 min centrifugation at 26000 g. For denaturation, 35 mg/ml of wet purified inclusion bodies was solubilized in buffer containing 6 M guanidinium hydrochloride (Gnd-HCl) as described previously [19]. The cell lysate containing denatured rhBMP-2 was extensively dialyzed to remove dithiothreitol (DTT) from the buffer then stored at −80°C.

### 2.2. Refolding and Purification of rhBMP-2 Protein

*In vitro* refolding and purification of native rhBMP-2 were performed with minor changes, as already described [14, 19]. 10 mg of total denatured protein was dissolved in 10 ml renaturation buffer containing 0.5 M Gnd-HCl, 50 mM Tris.HCl (pH 8.5), 0.75 M 2-(cyclohexylamino) ehanesulfonic acid (CHES), 5 mM EDTA, 2 mM reduced glutathione, and 1 mM oxidized glutathione at 1 mg/ml concentration. After 72 h of incubation at 15°C, the refolding mixture was diluted with an equal volume of 8 M urea; 20 mM Tris-HCl (pH 8.0), immediately passed through a 0.22 *μ*m filter (Millex-GP, Merck Millipore Ltd) and applied to 5 ml Hi-Trap heparin-sepharose column (GE Healthcare Bio-Sciences AB, Upsala, Sweden). The column was equilibrated with 5 column volumes of binding buffer containing 4 M urea, 20 mM Tris-HCl (pH 8.0). The monomeric and dimeric forms of rhBMP-2 were eluted in a two-step NaCl gradient buffer containing 350 and 500 mM, respectively. The eluted fractions were concentrated to about 1 mg/ml repeatedly by centrifugation using 5000 MWCO spin column (Vivaspin, Vivascience, Germany) and the buffer exchanged to 50 mM MES (pH 5.0), stored at −80°C to restore the biological activity.

### 2.3. Analytical Methods and Tests for Biological Activity of rhBMP-2

The protein concentration was determined using a Pierce BCA protein assay kit (Thermo Scientific). To test the quality of purified protein, 1 *µ*g of both monomeric and dimeric forms of rhBMP-2 protein were analyzed by gel electrophoresis using precast NuPAGE 4–12% Bis-Tris gels (Invitrogen) under reducing and nonreducing conditions. The gels were stained with Coomassie Brilliant Blue. To test the biological activity of rhBMP-2 protein, alkaline phosphatase induction (ALP) in C2C12 cells was measured according to procedures described by others [14, 19] and a colorimetric technique. Briefly, 1.2 × 10^5^ C2C12 cells were cultured in Dulbecco's Modified Eagle's Medium (DMEM) (Invitrogen) supplemented with 10% FBS and penicillin-streptomycin (P/S) (Sigma-Aldrich) per well in a 24-well plate at 37°C in a humidified incubator with 5% CO_2_ overnight. On the next day, the medium was replaced with 2% FBS followed by treatment of cells with rhBMP-2 protein in different dose concentrations (0, 0.25, 0.5, 0.75 1.0, and 1.25 *μ*g/ml). Standard rhBMP-2 protein was obtained from eBioscience, Inc. (http://www.eBioscience.com) and used as a positive control. After 72 h, the cells were washed with phosphate buffered saline (PBS) pH 7.4, lysed in RIPA buffer (Thermo Scientific), and the alkaline phosphatase activity of cell lysates was measured as described [14] using p-Nitrophenyl phosphate (pNPP) (Sigma-Aldrich) as a substrate for enzyme activity. The expression of alkaline phosphatase activity was also evaluated by a colorimetric staining technique in C2C12 cells treated with different doses of rhBMP-2 protein. After 3 days, the cells were washed with PBS and fixed with a citrate-acetone-formaldehyde solution for 30 s and stained for 30 min at room temperature using an Alkaline Phosphatase Leukocyte kit (Sigma-Aldrich).

In order to determine *in vitro* mineralizing effect of rhBMP-2, C2C12 cells were cultured in a 12-well plate with differentiation medium containing phenol-free minimum essential medium (MEM) supplemented with 15% FBS, 1 mM *β*-glycerophosphate, 1 mM ascorbic acid and antibiotic treated with either standard rhBMP-2 (eBioscience, Inc.) and purified bioactive rhBMP-2 (0.25 *μ*g/ml) and PBS as control. After 20 days, cells were washed with PBS, fixed with 50% ethanol and 18% formaldehyde, and stained with alizarin red and von Kossa for detection of microcalcification (IHC World).

### 2.4. Syngeneic Murine Model of Human Breast Cancer

Animal studies are performed under UT Southwestern Institutional Animal Care and Use Committee (IACUC) approved guidelines and protocols. Implanting human breast cancer cells in nude mice developed a syngeneic murine breast tumor model. Five to six weeks old female nude mice (NCI Ath/nu) were purchased from Charles River and housed under pathogen-free condition. The MDA-MDA-231 human breast cancer cell line was obtained from American Type Culture Collection (ATCC) and maintained in DMEM 10% FBS and 1% P/S at 37°C in a humidified incubator with 5% CO_2_. After trypsinization, ∼2.5 × 10^6^ MDA-MB-231 cells in a 100 *μ*l volume of ice cold PBS mixed with equal volume of Matrigel (Corning) were injected either unilaterally or bilaterally into the mammary fat pad at the base of the nipple. Four days after inoculation of tumor cells, the animals with a single tumor were injected intraperitoneally (IP) with PBS as vehicle or bioactive rhBMP-2 (15 *μ*g) for one time or two times on a weekly basis. In order to compare the systemic verses nonsystemic effects of rhBMP-2 on breast tumor calcification, the animals with bilateral tumors were treated with direct injection of PBS or rhBMP-2 into tumor in different doses (2.5, 7.5, and 15 *μ*g/tumor) on a weekly basis for two times.

### 2.5. CT Imaging of Breast Tumor Microcalcification *In Vivo*

Once the tumors reached 2 cm, the animals were anesthetized using 2% isoflurane/balanced with O_2_. Imaging was performed on a Siemens Inveon Multimodality PET/CT system for CT at medium magnification, 80 kVp, 500 *μ*A, and 130 ms exposure. CT images were reconstructed using the Feldkamp algorithm with a Shepp-Logan filter without down sampling.

### 2.6. PET/CT Imaging

One hour before imaging, animals were injected with approximately 150 *µ*Ci of ^18^F-NaF via tail vein, then scanned at 15 min postcontrast using list-mode PET centered at the tumor site, and a medium magnification CT was performed centering on the breast tumor sites using the Siemens Inveon Multimodality PET/CT. The animals were anesthetized using 2% isoflurane for the duration of the PET/CT scans. PET images were reconstructed using the 3D Ordered Subset Expectation Maximization (3D-OSEM) algorithm into one frame, and CT images were reconstructed using the Feldkamp algorithm and a Shepp-Logan filter. PET and CT images were coregistered using the Inveon Research Workplace (IRW) software (Siemens).

### 2.7. Histological Analysis of Breast Tumors

The tumors were harvested after PET/CT imaging, fixed overnight using 10% neutral buffered formalin (NBF) and embedded in paraffin. The FFPE tumor sections were prepared and loaded onto glass slides for staining with hematoxylin-eosin (H&E). Some slides were also prepared and stained with von Kossa for the detection of the presence of microcalcifications within the tumor using a von Kossa Stain Kit (IHC World).

## 3. Results

### 3.1. Recovery and Confirmation of Biological Activity of Purified rhBMP-2

**Figure 2:**
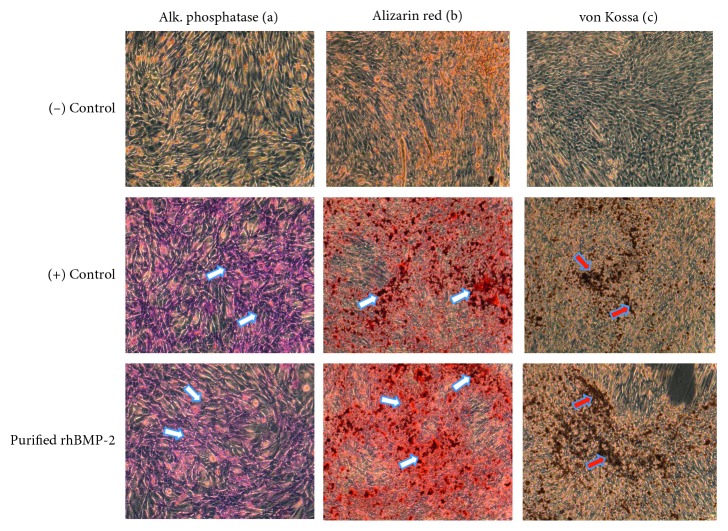
Microscopic detection of alkaline phosphatase activity and mineralization of C2C12 cells induced by rhBMP-2: microscopic detection of alkaline phosphatase in C2C12 cells (a) in presence and absence of rhBMP-2. The differentiation of C2C12 cells to osteoblast-like cells was achieved in differentiation medium for 21 days. The mineralization of differentiated cells induced by rhBMP-2 was detected using alizarin red (b) and von Kossa staining (c) as compared to controls treated with PBS. Magnification = 200x.

The bacterial expression construct of rhBMP-2 encoding a 13 kDa mature protein (Figure 1) and purification of protein were performed as described in Materials and Methods. The quality of rhBMP-2 protein samples (both monomers and dimers) was analyzed by SDS-PAGE gel electrophoresis followed by Coomassie blue staining. As shown in Figure 1, lanes 1 and 2 (monomer) and 3 and 4 (dimer) show samples with the reducing agent compared with the same samples in the absence of reducing agent (lanes 5–8). Our results showed the remaining residual host protein eluted together with 11 kDa protein using buffer containing 350 mM NaCl. In contrast, with an increase of salt concentration (500 mM NaCl) in elution buffer, the purity of eluted rhBMP-2 dimer was above 95% when analyzed under nonreducing gel electrophoresis shown in lane 7 and 8 (Figure 1). In order to test whether the purified rhBMP-2 is biologically active, we used an ALP assay. ALP is an extracellular enzyme produced during osteoblast differentiation and tissue mineralization [20]. Treatment of nonosteogenic C2C12 cells (myoblast) with rhBMP-2 and measurement of ALP activity have been used as a marker to determine the mineralizing effect of rhBMP2 in different tissues [14]. In this study, different doses of purified rhBMP-2 protein expressed in bacteria were used in C2C12 cells to determine the alkaline phosphatase activity and mineralization effect of rhBMP-2 in cultured cells. Commercial rhBMP-2 was used as positive control. As expected, after 72 h of treatment with rhBMP-2, the C2C12 revealed a significant increase of ALP in both positive control and experimentally purified protein in treated C2C12 cell lysates (Figure 1) as well as in colorimetric detected assay in culture plate (Figure 1). In order to detect *in vitro* mineralizing effect of rhBMP-2, C2C12 cells were grown in differentiation medium and treated with rhBMP2. After 20 days in culture, microscopic mineralization was detected by alizarin red and von Kossa staining (Figures 2 and 2), and compared to vehicle, there was nondetectable calcification when treated with PBS (Figure 2). The results indicate and confirm that the rhBMP-2 protein is present as intact dimeric protein and is biologically active for downstream application.

### 3.2. Syngeneic Human Breast Tumor in Murine Animal and PET/CT Imaging

**Figure 3:**
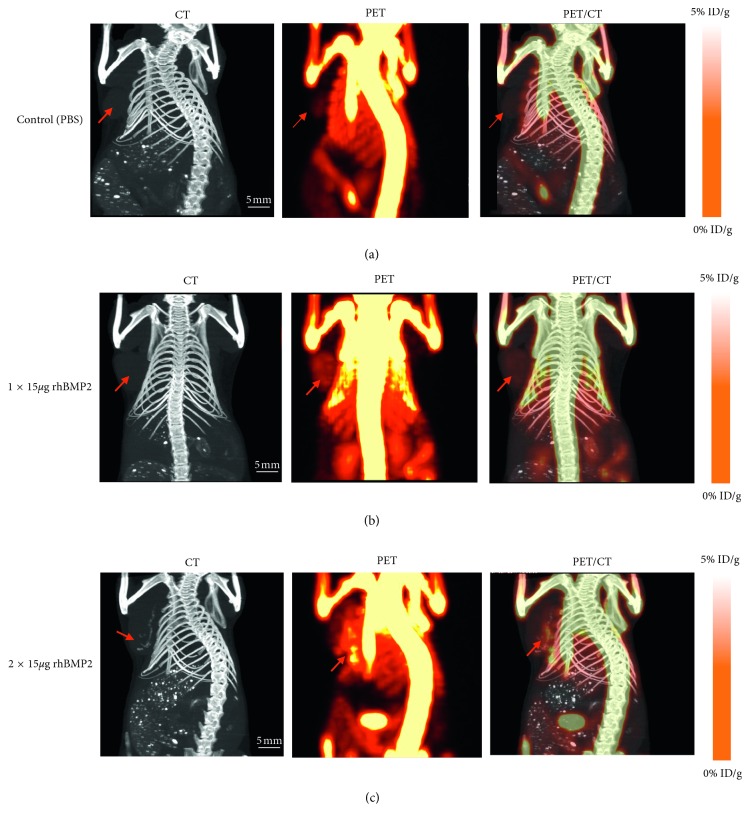
CT and PET/CT imaging of induced microcalcifications by rhBMP-2 in nude mice bearing MDA-MB-231 syngeneic tumors (a–c): the CT mages (left), PET (middle), and PET/CT fusion images (right). The CT images indicate that the animals injected IP with 15 *μ*g rhBMP-2 for two weeks (c) macroscopically induced higher level of microcalcifications by rhBMP-2 with significantly higher radiotracer (^18^F-NaF) uptake compared to a single dose (b). There was nondetectable microcalcification or no uptake of radiotracers by control animals injected with PBS as vehicle (a).

**Figure 4:**
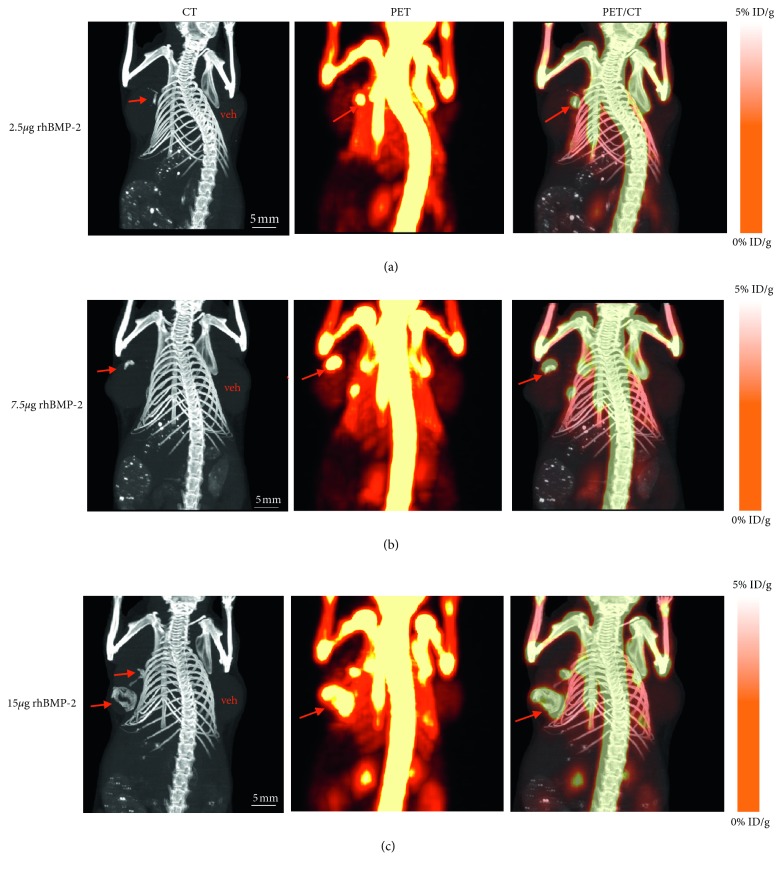
Macroscopic detection of macrocalcifications induced by direct injection of rhBMP-2 into MDA-MB231 syngeneic tumor: both CT imaging and PET imaging indicate that, in comparison to systemic, nonsystemic injection of rhBMP-2 into tumor induced microcalcification in breast tumor was dose dependent, and the uptake of radiotracer (^18^F-NaF) was significantly higher (left tumors in (a)–(c)) than the IP injection of rhBMP-2. There was nondetectable microcalcification or radiotracer uptake by control tumors (right tumors in a–c).

In this study, inoculation of MDA-MB231 cells with Matrigel into the mammary fat pad of nude mice developed tumor reproducibly. The growth of tumors in all animals was successfully monitored each week and continued for a 4-week period. When the tumors reached about 2 cm in diameter, the animals were briefly anesthetized with isoflurane and imaged with CT. Microcalcification of breast tumors that treated with rhBMP-2 either as IP or directly injection into tumors was detected by CT. The results indicate that the role of rhBMP-2 to induce microcalcification in breast tumor is both dose and time dependent when injected as IP (Figures 3 and 3) or directly injected into the tumors (Figures 4–4). In control animals that were injected with PBS either IP or directly into tumor, none of the tumors imaged by CT revealed microcalcification. Our CT results also showed that the animals injected IP with higher dose of rhBMP-2 (15 *μ*g) twice weekly (Figure 3), the tumors revealed higher levels of microcalcifications when compared to those injected with a single dose (Figure 3). In contrast to IP injection, the animals with two bilateral tumors with direct injection of rhBMP-2 into their tumors, exhibited higher microcalcifications detected by CT. Also, the results indicate that microcalcification induced by direct injection into tumor is dose dependent (Figures 4–4). In order to determine the type of calcification, i.e., hydroxyapatite or calcium oxalate (malignant tumor vs. benign respectively), the animals were intravenously injected with ^18^F-NaF as indicated in Materials and Methods to target hydroxyapatite and the tumors imaged by PET/CT. The uptake of radiotracer ^18^F-NaF by calcium deposits within the tumor induced by rhBMP-2 was significantly high and similar to bone uptake detected by PET imaging (Figures 3 and 4–4). The results support the specificity of radiotracer ^18^F-NaF in binding to hydroxyapatite, phenotypically to discriminate the malignant tissue from noninvasive benign tumor.

### 3.3. Histological Confirmation of Microcalcification Patterns in the Tumor Tissue

**Figure 5:**
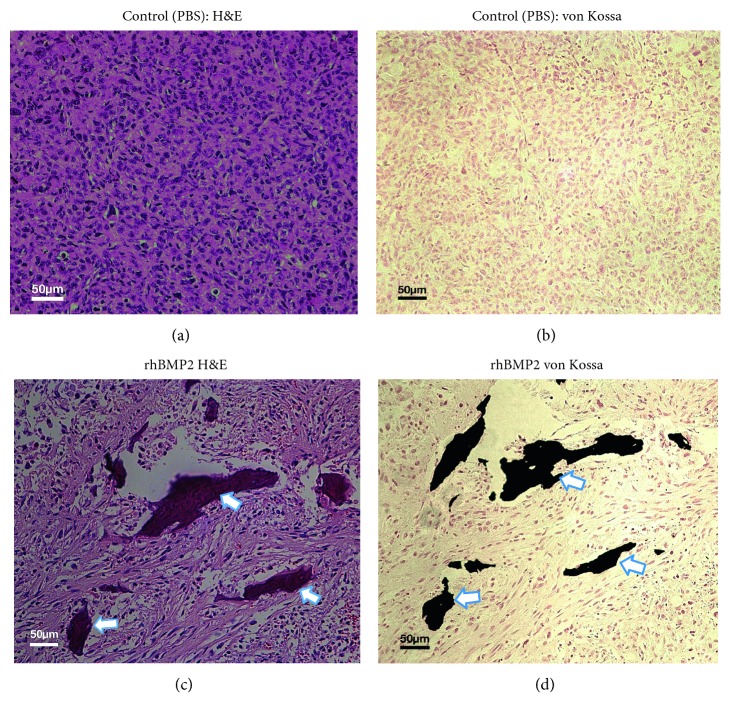
Histological analysis of post‐PET/CT imaging of MDA-MB231 syngeneic tumors: the tumor sections prepared from NBF fixed tissue stained with H&E (a, c) and von Kossa (b, d). Animals injected with BPS as vehicle (a, b) and high doses of rhBMP-2 (15 *μ*g) as IP or direct injection into tumors revealed microcalcifications stained with H&E and von Kossa (c, d). There was nondetectable microcalcification in tumor sections prepared from control tumors (a, b). Magnification = 200x.

Histological analysis was performed for further evaluation of calcium deposits detected by PET/CT imaging of breast tumors. The tissue specimens prepared from the rhBMP-2 treated group strongly showed microcalcifications when stained with H&E (Figure 5(c)) and von Kossa (Figure 5(d)) indicating the presence of calcium phosphate or hydroxyapatite reactive with silver nitrate. In comparison to the rhBMP-2 treated group, there was nondetectable microcalcification in control tumors injected with PBS as shown in Figures 5(a) and 5(b) respectively.

## 4. Discussion and Conclusions

In this study, we have developed a murine breast tumor microcalcification model using rhBMP-2 as a stimulatory factor and validated the model for its applicability on two imaging modalities: breast tumor detection (CT) and characterization of tumor features (^18^F-NaF/PET).

Our results indicate that nude mice with syngeneic human breast tumors that received rhBMP-2 either by IP injection or direct intratumoral injection exhibited microcalcifications detected by CT. The induction of microcalcifications by rhBMP-2 in this study was consistent with the previously results reported by Liu et al. that showed that rhBMP-2 induced microcalcification in a syngeneic rat breast tumor model [14]. On the basis of CT images, we were also able to show the extent of microcalcifications induced by rhBMP-2 was significantly higher when animals were injected with a high dose of rhBMP-2 after either IP injection or direct injection into tumors. The dose dependence of the microcalcifications induced by rhBMP-2 in our study was similar to the results reported in the syngeneic breast cancer model described in rats [14]. Similarly, an *in vitro* study also showed that MCF-7 cells treated with different doses of rhBMP-2 dose showed dose dependent increased microcalcifications detected by von Kossa staining [21]. Interestingly, in our study, we found that mice treated with intratumoral injections of rhBMP-2 showed significantly higher microcalcifications on CT images. We suggest that the more extensive microcalcifications induced by direct injection of rhBMP-2 may be caused locally by the increased duration of biologically active rhBMP-2 in the tumor. The detection of microcalcifications by high-resolution CT in this study was further characterized by PET imaging using ^18^F-NaF, which is commonly used as PET imaging agent that specifically binds to hydroxyapatite in bone. Our PET/CT images showed the uptake of ^18^F-NaF in the microcalcifications was significantly higher in tumors that were treated with higher doses of rhBMP-2. Based on CT and PET/CT imaging, we did not detect any microcalcifications on CT or radiotracer uptake by control tumors in absence of rhBMP-2 after 4 weeks of sham treatment. Our data were also consistent with previously reported studies with using rhBMP-2 to induce microcalcifications in the syngeneic breast tumor in rat model [10, 14]. In recent studies, Wilson et al. [18] and Felix et al. [17] subcutaneously injected MDA-MB231 cells in the right hind limb of mice followed by intravenous injection of contrast agents ^18^F-NaF for PET/CT and ^99m^Tc-MDP for SPECT imaging showing an uptake of radiotracers by these tumors. In their studies, the uptake of radiotracers by the tumors was lower than the uptake in bone, most likely due to the spontaneous generation of calcium complex in tumor that causes the uptake of radiotracers. In contrast, we found that none of the control breast tumors treated with PBS in the absence of rhBMP-2 revealed nondetectable microcalcifications by CT or radiotracers uptake by PET/CT imaging and even by histological analysis of tumors using von Kossa stain.

In conclusion, the syngeneic human breast cancer model that was created in murine animals can be used to assess the ability of targeted radiotracers to detect microcalcification in breast tumors and also to discriminate between the two different types of microcalcifications present in human breast cancers. In addition, we may also use this model for targeting the tumor using nanoparticles for delivery of therapeutic agents for either more effective treatment and/or minimizing any possible systemic toxicity of these agents.

## Data Availability

The data used to support the findings of this study are available from the corresponding author upon request.
